# Supplementary material for: Ginsenosides Restore Lipid and Redox Homeostasis in Mice with Intrahepatic Cholestasis through SIRT1/AMPK Pathways
Source: Nutrients. 2022 Sep 22;14(19):3938. doi: 10.3390/nu14193938 (PMC9571347; doi:10.3390/nu14193938)
Supplement: Supplementary file 1 [file nutrients-14-03938-s001.zip › nutrients-1880354-supplementary.pdf]

Supplementary Table S1. Primer sequence used in real-time RT-PCR

Name	Forward (5′ to 3′)
Mu-actin-F	GGCTGTATTCCCCTCCATCG
Mu-actin-R	CCAGTTGGTAACAATGCCATGT
Mu-CYP7A1-F	AGGCATTTGGACACAGAAG
Mu-CYP7A1-R	ACAGATTGGAGGTTTTGCAT
Mu-CYP27A1-F	TGAGGAAGAAAGAGGCTGA
Mu-CYP27A1-R	CAGAGCCGAATGGATGTAG
Mu-MRP2-F	TGACAACCTGAGCATAGGG
Mu-MRP2-R	TGCACTGGGAGAACTCG
Mu-BSEP-F	GTTCACGGAGCTTGAGTTG
Mu-BSEP-R	AAAAGCAGCCACTGTTCG
Mu-NTCP-F	GAAGTCCAAAAGGCCACA
Mu-NTCP-R	TGCCCACATTGATGACAG
Mu-SREBP-1-F	GCTGTTGGCATCCTGCTAT
Mu-SREBP-1-R	CTGGAAGTGACGGTGGTTC
Mu-FAS-F	CCAGTCGTGAAACCATACC
Mu-FAS-R	TCTTGCCCTCCTTGATGT
Mu-SCD1-F	TGTGCTAGATGGGATGGAG
Mu-SCD1-R	GCCTGGGGTCTTTGGTA
Mu-ACC1-F	TCACGCCACCTTGTCAG
Mu-ACC1-R	GGGGAGTCACAGAAGCAG
Mu-ChREBP-F	TCAGGGGATCTCAACTCCA
Mu-ChREBP-R	CGTCGGTTCTCCATCTTGT
Mu-HMGCR-F	GCCTTCCTTTTGGCTGT
Mu-HMGCR-R	GCTTTCTTTGAGGTCACGA
Mu-ACC2-F	CACCGAGTTCCTTGTGGTT
Mu-ACC2-R	GGGTCTCATCTGGCGTTC
Mu-PPARα-F	CATTTCTCCTTGGCGTGT

Mu-PPARα-R	CCTCAGACCTTGCTTTGG
Mu-CPT1-F	CCCAGTCAGATTCCAACC
Mu-CPT1-R	TCACCAAAATGACCTAGCC
Mu-HSL-F	CCGCCAGTTCCCTCTTT
Mu-HSL-R	CGACAGCACCTCAATCTCA
Mu-ATGL-F	ACTGCTGGGTCTCTTCTGC
Mu-ATGL-R	ACTTGGAGCTTGGCTGGT
Mu-CES1-F	GTTCGGTGTCCCATCTGT
Mu-CES1-R	GAGTTCATCAGCGTGGTCT
Mu-SIRT1-F	ACTGGAGCTGGGGTTTCT
Mu-SIRT1-R	CTTGAGGGTCTGGGAGGT
Mu-AMPK-F	TGTGGCTGGGTGTGTAAA
Mu-AMPK-R	GGCTGTGTGTGGCATTC
Mu-Nrf2-F	GATGGACTTGGAGTTGCC
Mu-Nrf2-R	CCTTCTGGAGTTGCTCTTG
Mu-HO-1-F	ACAGCCCCACCAAGTTC
Mu-HO-1-R	GGCGGTCTTAGCCTCTTC
Mu-GCLM-F	ATCAGTGGGCACAGGTAAA
Mu-GCLM-R	AGCAGTTCTTTCGGGTCA
Mu-GCLC-F	GGCATCGGAGAGGAGAA
Mu-GCLC-R	CAGGCTTGGAATGTCACC
Mu-NQO1-F	TCAGCCAATCAGCGTTC
Mu-NQO1-R	CTCCTTCATGGCGTAGTTG
